# Mechanism elucidation of cell‐selective fluorescent probes

**DOI:** 10.1002/smo2.70025

**Published:** 2025-11-14

**Authors:** Sourav Sarkar, Young‐Tae Chang

**Affiliations:** ^1^ SenPro Inc. Pohang Republic of Korea; ^2^ Department of Chemistry Pohang University of Science and Technology (POSTECH) Pohang Republic of Korea

**Keywords:** bioimaging, fluorescent probes, live‐cell distinction, targeted therapeutics

## Abstract

Cell‐selective fluorescent probes have emerged as essential tools for live‐cell imaging, enabling the differentiation of specific cell types within complex biological systems. Unlike traditional antibody‐based methods that target extracellular proteins, small‐molecule probes can access intracellular environments and exploit diverse biochemical features for selective retention or activation. This perspective categorizes the mechanisms of cell selectivity into five principal strategies: Protein‐oriented, carbohydrate‐oriented, lipid‐oriented, gating‐oriented, and metabolism‐oriented live‐cell distinctions. Each class capitalizes on a unique cellular trait ranging from protein expression and membrane composition to transporter activity and metabolic enzyme presence. We discuss representative examples of each mechanism, outline a decision‐tree workflow for elucidating a new probe's mode of action, and highlight how understanding these mechanisms is critical for both basic biological research and therapeutic probe design. Looking ahead, the development of such mechanism‐informed cell‐specific probes holds promise for advancing precision cell targeting in biomedical applications.

## Graphical abstract

This perspective outlines five mechanistic strategies—Protein‐oriented, carbohydrate‐oriented, lipid‐oriented, gating‐oriented, and metabolism‐oriented live‐cell distinctions—for achieving cell‐selective fluorescence in live‐cell imaging. By classifying probes based on their interactions with proteins, carbohydrates, lipids, transporters, or metabolic activities, the article provides a decision‐making framework for mechanism elucidation and highlights emerging logic‐gated designs for enhanced specificity in biological and diagnostic applications.

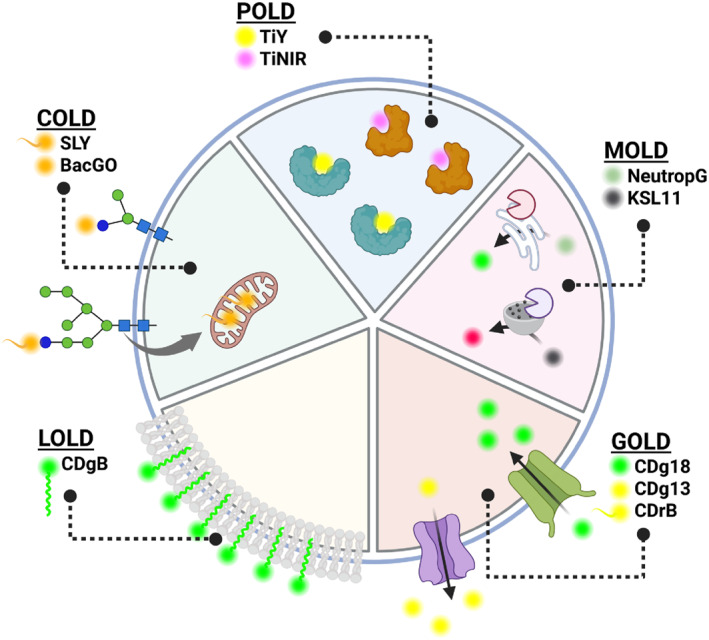

## 1 INTRODUCTION

**FIGURE 1:**
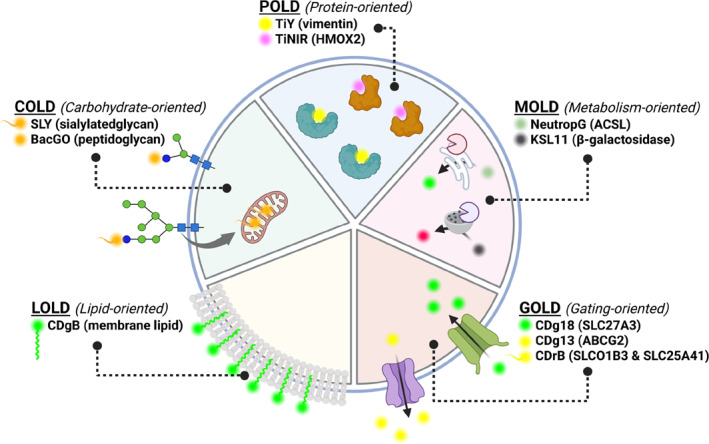
Overview of the five major strategies for cell‐selective fluorescent probes. In Protein‐oriented live‐cell distinction and Carbohydrate‐Oriented live‐cell distinction, probes bind selectively to intracellular proteins or cell‐surface carbohydrates unique to certain cells, leading to preferential retention. Lipid‐oriented live‐cell distinction probes target differences in membrane lipid composition or properties, accumulating in cells with distinctive membranes. Gating‐oriented live‐cell distinction probes leverage transporter proteins as “gates,” using differential uptake or efflux to concentrate the probe in one cell type over another. Metabolism‐Oriented live‐cell distinction probes are enzymatically converted or trapped in specific cells due to unique metabolic activities.

Distinguishing specific cell types in complex biological systems is crucial for understanding and manipulating biological processes. Traditional methods rely on antibodies binding to cell‐surface biomarkers, but these are limited to extracellular targets.^[1]^ Small‐molecule fluorescent probes offer a complementary strategy: they can permeate cells and target intracellular differences, enabling live‐cell distinction based on various biochemical features.[^2^, ^3^, ^4^] Such probes can be retained or activated in particular cells by different mechanisms—for example, by binding unique biomolecules, accumulating via specific transporters, or undergoing selective metabolism. Based on these mechanistic principles, researchers have categorized cell‐selective fluorescent probes into several strategies: Protein‐Oriented (POLD), Carbohydrate‐Oriented (COLD), Lipid‐Oriented (LOLD), Gating‐Oriented (GOLD), and Metabolism‐Oriented (MOLD) live‐cell distinction (Figure 1).^[5]^ Each strategy exploits a different aspect of cellular biology to achieve selectivity, and understanding these mechanisms is key for elucidating how a new probe works and for guiding the design of future probes.

## 2 UPTAKE MECHANISMS OF CELL‐SELECTIVE FLUORESCENT PROBES

### 2.1 Protein‐oriented live‐cell distinction

POLD probes achieve selectivity by binding to specific protein targets that are predominantly or uniquely expressed in the target cell type.[^6^, ^7^] The probe's affinity for a particular protein (such as an enzyme, receptor, or structural protein) causes it to accumulate in cells where that protein is abundant, thereby “holding” the probe in those cells. This is analogous to a classic targeted labeling approach, using a small molecule instead of an antibody. If a fluorescent compound discovered via screening is retained only in certain cells, one hypothesis is that it binds to a protein biomarker enriched in those cells.

**FIGURE 2:**
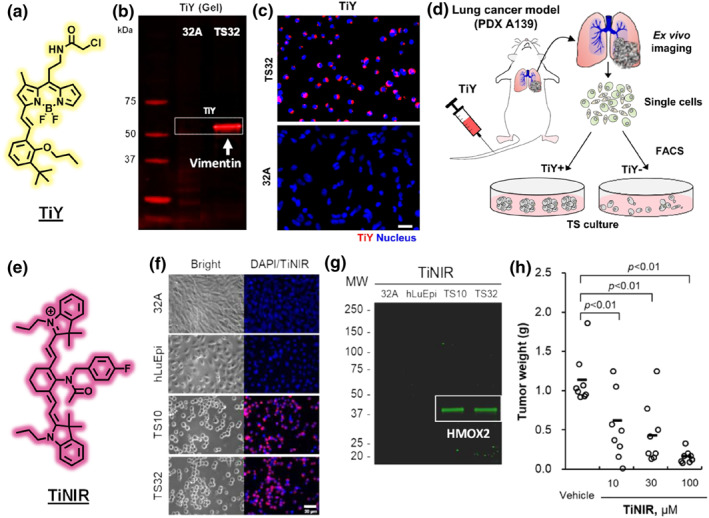
(a) Structure of a TIC selective probe TiY. (b) Fluorescence scan of gel subjected for 1D SDS‐PAGE run with lysate extracted from TiY prestained 32A and TS32, respectively. (c) TiY selectively stain TS32 cells over its isogenic counterpart control 32A cells. Scale bar, 250 μm. (d) Schematic diagram of the experimental procedure for TiY dependent sorting to assess the TICs. (e) Structure of a TIC selective probe TiNIR. (f) Fluorescence images of 32A, hLuEpi, TS10, and TS32 cells after being stained with TiNIR and DAPI. Scale bar, 30 μm. (g) SDS‐PAGE image of the proteome labeled with TiNIR in negative (32A and hLuEpi) cells and positive (TS10 and TS32) cells (right). (h) Inhibition of tumor by TiNIR in tumor‐bearing mice. 10, 30, and 100 μM of TiNIR was injected into the tail vein (200 μL/mouse) every couple of days until day 25. ○, individual tumor; −, mean of group; *N* = 4. The paired two‐tailed Student's *t*‐test was used to determine the significances of differences between groups. Reproduced from Ref.[^8^, ^9^] Copyright (2023) IVY Spring and (2019) American Chemical Society, respectively. TIC, tumor‐initiating cell.

A notable example is the tumor‐initiating cell (TICs) probe TiY, a fluorescent molecule that binds to vimentin, an intermediate filament protein upregulated in epithelial‐to‐mesenchymal transition (EMT) and certain cancer stem‐like cells (Figure 2a,b).^[8]^ By targeting vimentin, TiY selectively stains TICs while sparing differentiated tumor cells or normal cells (Figure 2c). This protein‐oriented mechanism enabled the identification and isolation of TICs using fluorescence imaging and sorting (Figure 2d). TiY was used to inhibit tumor formation in a lung cancer mouse model at a higher concentration. Another example is a near‐infrared probe TiNIR targeting heme oxygenase‐2 (HMOX2) (Figure 2e–g). The probe was used to track and treat lung TICs in a tumor bearing mouse model (Figure 2h).^[9]^ These cases illustrate how POLD probes can serve as fluorescent markers for cell identity when a defining protein biomarker is known or discovered. POLD strategies leverage the high specificity of protein‐ligand interactions, but they depend on the existence of a suitably unique protein target in the cell type of interest (a potential limitation when cell‐type‐specific proteins are unknown or absent in accessible form). In practice, if a new probe is suspected to be POLD, researchers will often perform target identification experiments (e.g., affinity pull‐down and mass spectrometry) to find the protein partner, confirming that binding to that protein underlies the selective staining.[^10^, ^11^]

### 2.2 Carbohydrate‐oriented live‐cell distinction

COLD probes selectively recognize carbohydrate structures (such as glycans or polysaccharides) that differ between cell types.[^12^, ^13^] Many cell types have distinct cell‐surface carbohydrates (e.g., glycoproteins or glycolipids with unique sugar moieties) which can serve as markers.^[14]^ A fluorescent probe designed to bind a specific sugar epitope or to react with a particular carbohydrate can thus label only the cells presenting that glycan. Unlike proteins, carbohydrates are often extracellular, so COLD probes typically target cell‐surface or extracellular matrix components.^[15]^ This approach extends selectivity beyond protein antigens to the often‐overlooked glycomic differences between cells (such as differential sialylation or glycosaminoglycan expression).

**FIGURE 3:**
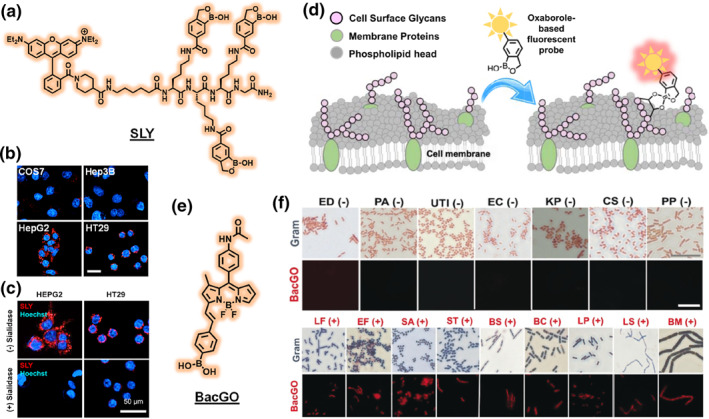
(a) Structure of sialylated glycan selective cancer cell probe SLY. (b) Fluorescence imaging of COS7, Hep3B, HepG2, and HT29 cells incubated with SLY. Scale bar, 20 μm. (c) Effect of sialidase on SLY staining in HepG2 and HT29 cells. Scale bar, 50 μm. (d) Mechanism of sialylated glycan recognition with SLY. (e) Structure of peptidoglycan selective gram positive bacteria probe BacGO. (f) Gram staining and BacGO based fluorescent staining of 16 bacterial strains (7 g negative, 9 g positive). Scale bar, 10 μm. Reproduced from Ref.[^16^, ^17^] Copyright (2025) American Chemical Society and (2019) Wiley‐VCH, respectively.

One classic demonstration of COLD is the use of boronic acid‐based fluorescent probes to target cell‐surface sialylated glycans. For instance, an oxaborole functionalized rhodamine probe, SLY, was shown to selectively bind to cells expressing the sialyl Lewis X and A antigens (specific to certain leukocytes and cancer cells) (Figure 3a).^[16]^ This probe exploits the covalent binding of the oxaborole to *cis*‐diol groups in sugars, effectively distinguishing cells by the presence of that carbohydrate marker (Figure 3b–d).^[18]^ The probe was used to detect cancerous regions within the mouse liver at cellular resolution. Other examples include fluorescent lectin‐mimicking molecules that stain bacterial cells by binding to unique cell wall sugars (e.g., peptidoglycan in Gram‐positive bacteria, as with probe “BacGO” in the literature) (Figure 3e).^[17]^ The probe was used to detect Gram‐positive bacterial infection in vitro and in vivo in a mouse model of keratitis (Figure 3f). While COLD strategies are less common than protein‐targeted probes in mammalian cell typing, they offer a powerful way to differentiate cells such as immune cell subsets or cancer cells based on glycosylation patterns. The challenge is designing small molecules with high specificity for a particular carbohydrate motif, but when successful, they can label cells in a manner analogous to carbohydrate‐targeting antibodies (lectins) but with the flexibility of synthetic dyes.

### 2.3 Lipid‐oriented live‐cell distinction

LOLD probes exploit differences in lipid composition or membrane properties between cell types. Cell membranes vary in their content of phospholipids, cholesterol, and other lipids, which can affect membrane fluidity, phase behavior, and the presence of microdomains. Certain cell types (e.g., different leukocytes, neurons vs. glia, or healthy vs. cancerous cells) and tissues have characteristic membrane lipid profiles.[^19^, ^20^] A LOLD probe is typically a lipophilic molecule that preferentially inserts into or interacts with membranes having a particular composition or biophysical property (such as a specific cholesterol level or degree of saturation in fatty acids).^[21]^ By “sensing” the membrane environment, the probe accumulates more in cells with the matching membrane characteristics, enabling selective staining without directly binding a protein or sugar.

**FIGURE 4:**
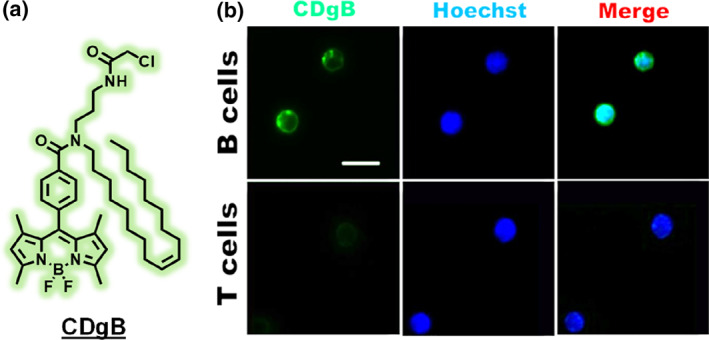
(a) Structure of B‐cell selective probe CDgB. (b) CDgB treated B and T cells isolated from the murine spleen. Scale bar, 10 μm. Reproduced from Ref.^[22]^ Copyright (2021) American Chemical Society.

A representative example of LOLD is the fluorescent probe CDgB, which was found to selectively stain B lymphocytes over T lymphocytes due to differences in their plasma membrane properties (Figure 4a,b).^[22]^ In this case, CDgB's uptake or retention correlates with membrane flexibility (fluidity) and possibly lipid composition, which differ between B and T cells. The B cells' membranes provide a favorable environment for CDgB, whereas T cell membranes do not, leading to selective labeling. This kind of mechanism might arise if, for instance, B cells have a higher cholesterol content or a distinct membrane domain that sequesters the probe. Other examples of LOLD probes could include dyes that highlight myelinated cells (rich in lipids) versus unmyelinated cells or probes that accumulate in adipocytes (fat cells) due to their lipid storage.[^23^, ^24^] LOLD strategies underscore that membrane lipidomics can be a basis for cell selectivity. One practical note is that LOLD probe interactions are often hydrophobic and less specific than lock‐and‐key protein interactions, so achieving high specificity can be challenging. Nonetheless, as shown by CDgB's B‐cell selectivity, carefully designed lipophilic probes can discern cell types based on membrane microenvironment differences that are invisible to protein‐targeted approaches.

### 2.4 Gating‐oriented live‐cell distinction

GOLD probes utilize transporter proteins (permeability gates) to achieve selective accumulation. Every cell type expresses a unique repertoire and level of membrane transporters—including solute carriers (SLC transporters that import nutrients and molecules) and ATP‐binding cassette transporters (ABC pumps that export substances)—which control the influx and efflux of various compounds.^[25]^ A GOLD probe is typically a molecule that either mimics an endogenous substrate of a transporter or coincidentally fits into a transporter's substrate range. If a target cell type highly expresses a particular uptake transporter (pump‐in) for which the probe is a substrate analog, that cell will accumulate the probe more than others. Conversely, if other cells express an efflux pump that actively removes the probe (pump‐out) while the target cell does not, the target cell will retain more probe. Thus, differences in transporter expression create a gating effect, concentrating the probe in one cell population over another. Notably, this mechanism does not require the probe to bind any intracellular target at all—the selectivity arises from cellular import/export dynamics.

**FIGURE 5:**
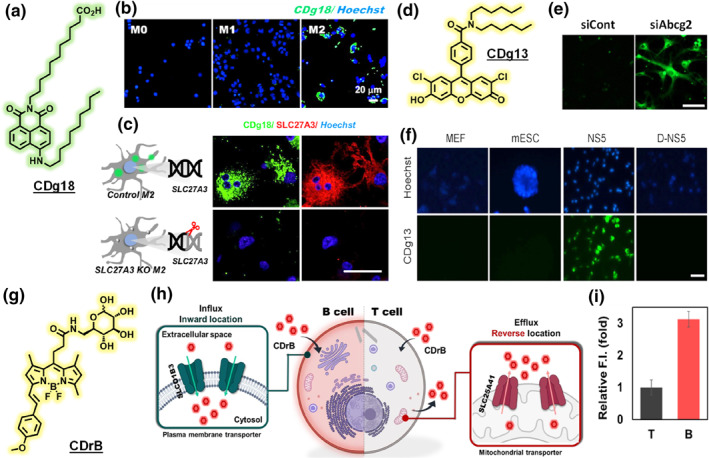
(a) Structure of M2 macrophage selective probe CDg18. (b) Selectivity of CDg18 toward M2 macrophages. Scale bar, 20 μm. (c) Fluorescence images of CDg18 (green), SLC27A3 antibody (red), and Hoechst (blue) of the control and Slc27a3 KO cells. Scale bar, 20 μm. (d) Structure of neural stem/progenitor cells (NSPCs) selective probe CDg13. (e) Control siRNA‐treated (siCont) and ABCG2‐knocked down (siAbcg2) D‐NS5 cells were stained with CDg13. Scale bar, 50 μm. (f) Live cells were stained with CDg13 and Hoechst 33,342. Scale bar, 10 μm. (g) Structure of B cell selective probe CDrB. (h) Proposed mechanism of B‐cell selective staining of CDrB. CDrB is taken up by SLCO1B3 expressed in both T and B lymphocytes. However, T cells, which overexpress SLC25A41 compared to B lymphocytes, actively efflux CDrB. (i) B cell selectivity of CDrB is shown with the relative fluorescence intensity of CDrB treated T and B lymphocytes collected from murine spleen. Reproduced from Ref.[^31^, ^32^, ^33^] Copyright (2023) American Chemical Society, (2016) Wiley‐VCH and (2024) MDPI, respectively.

Many GOLD probes have been developed by capitalizing on transport pathways for nutrients or metabolites.[^26^, ^27^, ^28^] For instance, fluorescent analogs of glucose, such as 2‐NBDG,^[29]^ are often taken up by cells with high glucose transporter (GLUT1) activity.^[30]^ Another striking example is CDg18, which was reported as the first M2 macrophage‐selective dye acting through a gating mechanism: it exploits fatty acid transporter differences in macrophage subtypes (Figure 5a,b).^[31]^ M2‐polarized macrophages express certain long‐chain fatty acid transporters such as SLC27A3 at higher levels, and CDg18 (an analog of a fatty acid) is taken up more by M2 cells, allowing fluorescent distinction of M2 versus M1 macrophages (Figure 5c). On the efflux side, probes such as CDg13 have been identified that selectively stain cells lacking certain ABC transporters (like ABCG2) (Figure 5d,e). Cells with high efflux activity pump these dyes out and remain dim, whereas cells with low efflux accumulate the dye and appear bright (Figure 5f).^[32]^ The probe was used to isolate neural stem/progenitor cells (NSPCs) from embryonic mouse brain. A particularly sophisticated GOLD mechanism was found in a recent study of a B‐cell selective probe CDrB: this probe's selectivity relies on two transporters working in opposition—SLCO1B3 (an uptake transporter bringing the probe in) and SLC25A41 (an efflux transporter pumping it out) (Figure 5g,h).^[33]^ Both T and B cells take up CDrB via SLCO1B3, but T cells have high SLC25A41 that actively exports the probe, whereas B cells do not—resulting in retention of the probe in B cells and effective discrimination between B and T lymphocytes (Figure 5i). This finding highlights how multiple gating factors can combine to create cell specificity. Overall, GOLD approaches have opened new avenues for probe development—virtually any differential transporter expression (across cell types or disease states) is an opportunity to design or discover a selective probe. Tools such as transporter inhibitor panels and CRISPR knockout libraries greatly aid in elucidating which transporter is responsible for a probe's cell‐selective uptake, reinforcing the central role of transporters in these mechanisms.^[34]^

### 2.5 Metabolism‐oriented live‐cell distinction (MOLD)

MOLD probes are designed to be activated or selectively retained by specific metabolic activities or enzymes present in the target cells. In this strategy, the probe often starts as a non‐fluorescent or masked form (e.g., a fluorogenic substrate or a cell‐permeable pro‐dye) that is chemically transformed by an enzyme or metabolic process unique to the cell of interest.[^35^, ^36^] Only in cells that have that enzymatic activity will the probe be converted into a fluorescent (or membrane‐impermeable) product, which then accumulates. This “metabolic trapping” mechanism results in selective illumination of cells with the corresponding metabolic profile, functioning much like an enzymatic assay performed in live cells. MOLD takes advantage of the fact that different cell types (or states, such as activated immune cells vs. resting) express different sets of enzymes—whether they be hydrolytic enzymes, oxidoreductases, transferases, etc.—and these differences can be harnessed for selectivity.

**FIGURE 6:**
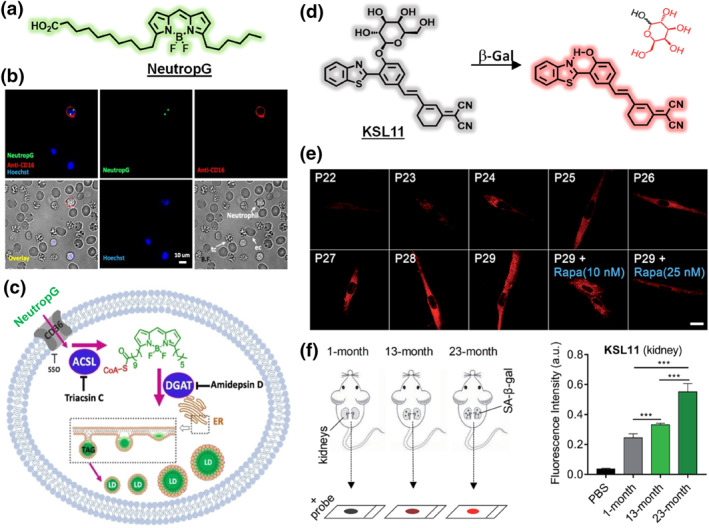
(a) Structure of neutrophil selective probe NeutropG. (b) Selectivity of NeutropG for neutrophils in whole blood without RBC lysis. NeutropG and APC anti‐CD16 were incubated with whole blood for 1 h followed by Hoechst for 10 min prior to imaging. Scale bar, 10 μm. (c) The proposed staining mechanism of NeutropG. (d) Structure of aging cell selective probe KSL11 and its enzymatic reaction with beta galactosidase. (e) Confocal fluorescence images of MRC5 cells incubated with KSL11 at various stages of replicative senescence (passage numbers ranging from 22 to 29), and senescent MRC5 cells (P29) incubated with Rapa for 3 days before labeling with KSL11. Scale bar, 10 μm. (f) Schematic diagram of kidneys changes with natural aging. Quantified fluorescence intensity of KSL11 in PBS and the kidney sections from 1‐, 13‐, 23‐month‐old C57 male mice after incubation with KSL11. Error bars represent the standard deviation (±S.D.) with *n* = 5. Reproduced from Ref.[^38^, ^39^] Copyright (2021) Wiley‐VCH and (2020) Royal Society of Chemistry, respectively.

A well‐known general example of MOLD is the viability/cytotoxicity indicator calcein AM—a nonfluorescent acetoxymethyl ester that is cleaved by ubiquitous intracellular esterases to release fluorescent calcein.^[37]^ Live cells with active esterases rapidly turn calcein AM into fluorescent calcein (trapped inside because it's now charged), whereas dead cells do not—thereby distinguishing live cells. While that example distinguishes live versus dead rather than specific cell types, it illustrates the principle of metabolic activation. For cell‐type selectivity, the key is to target an enzyme that is particularly active in one cell population. One recent success is the probe NeutropG, which was developed to specifically label neutrophils among white blood cells (Figure 6a,b).^[38]^ NeutropG is highly lipophilic and is believed to be metabolized by neutrophil‐specific enzymes involved in lipid uptake or storage, leading to a fluorescent product that accumulates only in neutrophils (Figure 6c). This allows imaging of active neutrophils in a mixed cell environment via a metabolism‐dependent turn‐on mechanism. The probe was used to quantify neutrophil levels in fresh blood samples with high accuracy. Another example is a “tandem‐lock” fluorescent reporter TNR1 that requires two enzymes (neutrophil elastase and cathepsin G) to both act before it fluoresces—effectively an AND‐gate probe that lights up exclusively in neutrophils undergoing a specific process (NETosis).^[40]^ Likewise, fluorescent substrates such as KSL11 for enzymes like β‐galactosidase have been used to identify senescent cells (which overexpress senescence‐associated β‐gal) (Figure 6d,e).^[39]^ Study on a naturally aging mice model suggests that the kidneys are the organs with highest susceptibility toward natural aging (Figure 6f,g). Also, probes activated by kinase,^[41]^ oxidase,^[42]^ and reductase^[43]^ have been proposed to differentiate cancer cells with unique metabolic signaling. These cases demonstrate the flexibility of MOLD: by linking probe activation to a hallmark enzymatic activity of a cell type, one can achieve highly selective staining.[^44^, ^45^] A challenge with MOLD is ensuring that the enzymatic reaction is specific and efficient enough in the target cells without occurring in others—but when a distinctive metabolic difference exists, MOLD probes can exploit it to great effect. As a bonus, the fluorescence turn‐on nature of many MOLD probes yields high contrast (low background in non‐target cells).

### 2.6 Others

Beyond the five mechanistic classes discussed here, environment‐sensitive probes that respond to polarity or viscosity changes also offer intriguing opportunities for cell‐selective imaging. Cancer cells, for instance, often exhibit altered membrane polarity and higher intracellular viscosity compared to normal cells, and polarity/viscosity‐sensitive fluorophores can harness these differences for selective visualization. Such approaches have been reviewed for polarity probes.[^46^, ^47^, ^48^, ^49^] On the viscosity side, probes mapping intracellular, mitochondrial or membrane viscosity have been employed to distinguish cancer cells from the normal ones.[^50^, ^51^] While these probes do not fit neatly into the current biochemical categories (POLD, MOLD etc.), they highlight the potential of microenvironmental properties as an axis of selectivity. Looking forward, combining environment‐sensing with established strategies—such as transporter gating or metabolic activation—may yield hybrid designs with even greater precision. These approaches underline the expanding landscape of probe design, where both biochemical and biophysical cues can be rationally exploited using suitable fluorophores to illuminate specific cell populations.[^52^, ^53^, ^54^]

## 3 MECHANISM ELUCIDATION WORKFLOW FOR NEW PROBES

**FIGURE 7:**
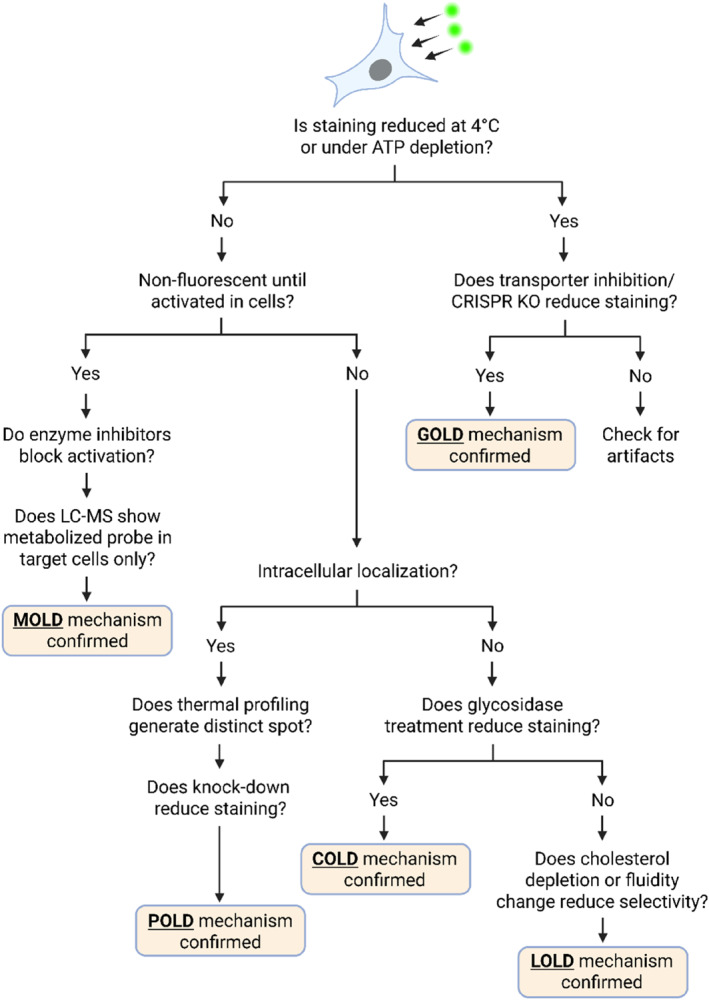
A decision logic flowchart to visualize the possibilities of live cell distinction.

When a novel cell‐selective fluorescent probe is discovered (for instance, via high‐throughput screening of a chemical library), elucidating its mechanism of selectivity is critical. Determining why a probe stains one cell type and not another will validate the probe's utility and guide further development. A logical, stepwise approach can be taken to identify the underlying mechanism, essentially working through the possible strategies (POLD, COLD, LOLD, GOLD, MOLD) like a decision tree (Figure 7).

### 3.1 Phenotypic characterization

First, confirm the basic phenotype of selectivity. Verify that the probe's selective staining is reproducible and truly cell‐specific (e.g., it consistently labels cell type A and not type B under various conditions). Identify where the fluorescence localizes (nucleus, cytoplasm, membrane?) and whether the probe is active (fluorogenic) or simply accumulative. These observations may give initial clues (e.g., membrane‐localized staining might hint at a membrane component or transporter mechanism).

### 3.2 Protein target identification (POLD hypothesis)

Investigate whether the probe binds to a specific protein that could explain its selectivity. Common techniques include affinity precipitation (attach the probe or an analog to beads and pull down binding proteins from cell lysates) and thermal stability shifts (to see if any protein is stabilized by probe binding).^[55]^ If these experiments identify a protein that is present in the stained cells but low/absent in unstained cells, the mechanism is likely protein‐oriented. For example, if a pulldown reveals that a probe binds strongly to an enzyme that is known to be overexpressed in cell type A, one can conclude that the probe is a POLD‐type stain for that enzyme. It is important to validate by loss‐of‐function: does knocking down or inhibiting that protein reduce probe uptake? Does overexpressing it in a negative cell induce staining? Answering these questions will confirm a POLD mechanism. If no unique protein partner is found (or if the identified protein is actually common to all cells), then a pure POLD mechanism may be ruled out, and other mechanisms should be examined.

### 3.3 Carbohydrate involvement (COLD hypothesis)

If the probe tends to stain cell surfaces or extracellular areas, consider a carbohydrate‐based mechanism. Perform experiments like competitive inhibition or removal of carbohydrates: for instance, pre‐treat cells with specific glycosidases to cleave certain sugars, or add excess soluble competing sugars/lectins, to see if the probe staining is diminished. If treating cells with, say, a sialidase enzyme abolishes the selective staining, it suggests that the probe was targeting a sialic acid‐containing epitope (consistent with COLD). Likewise, check if the probe fails to discriminate between cells when surface glycosylation is globally inhibited (using a metabolic inhibitor of glycosylation). A positive result would implicate a carbohydrate as the key target. Reference to known examples can guide what to test; for example, if the target cells are known to have a unique glycan (as B cells have sialyl Lewis X in certain differentiation stages), test that specifically. If none of these perturbations affect the staining, a COLD mechanism is less likely.

### 3.4 Membrane lipid dependency (LOLD hypothesis)

If the probe is very hydrophobic or localizes to membranes, test whether membrane composition or fluidity underlies the selectivity. One approach is to alter membrane properties and observe the effect on staining: for example, use methyl‐β‐cyclodextrin to extract cholesterol from membranes,^[56]^ or incorporate fatty acids to change membrane fluidity,^[57]^ and see if the probe's selectivity is lost or reduced. Another test is swapping the membranes, for example, creating hybrid cells or liposome models with different lipid make‐ups to determine where the probe prefers to partition. If the probe's selective uptake correlates with the presence of certain lipids (say, it only accumulates in cholesterol‐rich membrane domains and the target cell has more of those domains), that points to a LOLD mechanism. Additionally, comparing mutant cell lines with known differences in membrane lipid metabolism (for instance, a cell that cannot produce a certain lipid) can be informative. However, purely lipid‐based mechanisms can be hard to pin down; often they are confirmed by exclusion of other possibilities plus supportive evidence (like distinct biophysical behavior of the probe with membranes from target vs. non‐target cells).

### 3.5 Transporter gating tests (GOLD hypothesis)

To probe a gating‐oriented mechanism, determine if active transport processes are required for the probe's entry/exit. A simple indicator is to test whether energy dependence exists: for example, if you incubate cells at 4°C (where active transport halts) or treat with ATP‐depletion (like sodium azide), does the probe's uptake and selectivity disappear?^[68]^ If yes, it implies an active transporter might be involved (passive diffusion would be less temperature‐sensitive). Next, employ a transporter inhibitor panel—a set of broad‐spectrum or selective inhibitors for various uptake transporters (OATPs, GLUTs, amino acid transporters, etc.) and efflux pumps (P‐gp, MRPs, etc.). If a particular inhibitor (or combination) blocks the probe's selective staining, it suggests transporter's involvement. Modern approaches use CRISPR‐Cas9 libraries or CRISPR activation screens: for instance, do a genome‐wide knockout screen for cells that lose probe accumulation or an overexpression screen for cells that gain staining. In the B versus T cell example (CDrB probe), a targeted CRISPR activation screen of SLC transporters identified SLCO1B3 and SLC25A41 as key players.^[32]^ Generally, if the data point to a specific transporter (or a small set of them), one can confirm by directly manipulating that transporter: overexpress it in negative cells to see if they uptake the probe, or knock it out in positive cells to see if they lose staining. Such experiments solidify a GOLD mechanism. Notably, gating mechanisms may sometimes involve more than one transporter (as in the CDrB case), so one should check both uptake and efflux sides. If no transporter effect is seen (e.g., inhibitors and knockouts do not change probe distribution), then gating is likely not the primary mechanism.

### 3.6 Metabolic activation tests (MOLD hypothesis)

To test a metabolism‐oriented mechanism, evaluate whether enzymatic activity in the target cell is needed for the probe's fluorescence. A hallmark of MOLD probes is that fixed or dead cells typically will not show selective staining, since metabolism has stopped. Thus, fixing both target and non‐target cells after probe treatment can reveal if the difference persists (if it disappears upon fixation, a metabolic step might be required). Another approach is to use enzyme inhibitors: if you suspect a certain enzyme (say, a kinase, esterase, or oxidase) could be activating the probe, treat cells with a specific inhibitor for that enzyme. If the target cells lose fluorescence when the enzyme is inhibited, that is strong evidence for that enzyme's role. Broader approaches include comparing the metabolomic or biochemical profiles of the cells: target cells might convert the probe into a unique fluorescent product, which can be detected by techniques like LC‐MS. Indeed, analyzing cell extracts for probe metabolites can directly show whether a chemical transformation has occurred. For example, one might find that only in target cells the probe has been cleaved or conjugated (e.g., glucuronidated) into a new fluorescent species that is absent in other cells. If an enzyme‐mediated conversion is identified, confirm by knocking down that enzyme—the probe's selectivity should diminish. In summary, a positive identification of a unique enzymatic activation (or trapping) of the probe confirms the MOLD mechanism. If exhaustive testing finds no such differences, then metabolism may not be the key factor.

### 3.7 Integration and complex mechanisms

After these systematic tests, the evidence usually points to one primary mechanism. However, it is possible that a probe's behavior involves multiple mechanisms in concert. For instance, a probe might require a transporter to enter the cell (GOLD) and then be metabolized to remain trapped (MOLD), or it might bind a protein only after being metabolized. If initial results are confusing or point to two mechanisms, consider a dual mechanism—this can actually increase specificity by requiring two conditions. In such cases, you would need to demonstrate both components (e.g., show that disabling either the transporter or the enzyme partly reduces staining, but only disabling both eliminates it).

Ultimately, identifying the mechanism allows to classify the probe into POLD, COLD, LOLD, GOLD, or MOLD (or a hybrid thereof) and provides a mechanistic explanation for its cell selectivity. This investigative workflow is akin to debugging a circuit: by systematically toggling “inputs” (proteins, sugars, transport, enzymes) and observing outputs (probe staining), one deduces the logical circuit that produces the selective signal. Importantly, understanding the mechanism not only satisfies scientific curiosity but also is essential for validating the probe (ensuring it truly reports what we think it does) and for optimizing or generalizing the probe (e.g., can we modify it to improve binding or transport now that we know the key players?).

## 4 CONCLUSION AND FUTURE PERSPECTIVES

Over the past decades, fluorescent molecules have emerged as a valuable tool for studying sensing and optoelectronic applications, organelle biology, disease diagnosis, therapeutics, etc.[^59^, ^60^, ^61^, ^62^] In particular, cell‐selective fluorescent probes have enabled researchers to highlight specific cells amidst the diversity of tissues and cultures. In this perspective, we outlined the major mechanisms—POLD, COLD, LOLD, GOLD, and MOLD—by which these probes achieve selectivity, and discussed how one can systematically determine which mechanism is at play for a newly discovered probe. Each strategy offers unique advantages: protein‐oriented probes provide high specificity via biomarker targeting, carbohydrate‐ and lipid‐oriented probes can target non‐protein cell surface features, metabolism‐oriented probes give conditional activation with high contrast, and gating‐oriented probes tap into the rich variety of transporter biology to find novel selectivity axes. Equally important, each comes with its own challenges (e.g., identifying a truly unique target, or ensuring an enzyme reaction is specific enough, etc.), and thus a thorough mechanistic elucidation is necessary before claiming a probe is cell‐type‐specific (Table 1).

**TABLE 1:** Advantages and challenges of different live‐cell distinction mechanisms.

Mechanism	Advantage	Challenges
POLD	High specificity due to selective protein binding	Target identification is time‐consuming
COLD	Targets extracellular glycans only. No cell entry offers lower cytotoxicity	Carbohydrate structures are often similar between cell types, COLD probes are rare
LOLD	Can label live cells without enzymatic targets	Specificity can be lower due to nonspecific hydrophobic interactions
GOLD	Can label live cells without enzymatic targets	Depends on cell condition and metabolic state
MOLD	Fluorescence activation yields high contrast	Depends on cell state or culture conditions

Looking forward, new concepts and hybrid strategies are expected to expand this field. One promising direction is the design of multi‐input logic probes that require two or more conditions to be met—increasing specificity, like an electronic AND gate. We already see hints of this: the tandem‐enzyme activated probe for neutrophils required two proteases,^[38]^ and the CDrB probe effectively used an AND/NOT gate (presence of one transporter AND absence of another)^[32]^ to distinguish B from T cells. Future probes might be deliberately engineered to combine, say, a specific transporter uptake sequence with a metabolic activation step, so that only cells with both the transporter and the enzyme light up. This “gated activation” approach could yield ultra‐selective imaging agents for complex tissues (imagine a probe that only fluoresces in a particular immune cell subtype that both takes it up and then metabolizes it by a unique enzyme). Additionally, as our knowledge of cell‐specific metabolomes and transportomes grows (through single‐cell sequencing and proteomics), we can rationally design probe libraries biased toward certain mechanisms—for example, designing substrates for an enzyme known to be in a target cell, or scaffolds resembling the ligand of a transporter expressed only in a particular cell lineage. Machine learning and computational modeling might assist in predicting the chemical features that drive these interactions, accelerating the discovery of targeted probes.^[63]^ From a practical standpoint, integrating modern techniques such as CRISPR screening into the probe discovery pipeline will greatly enhance mechanism elucidation. Instead of treating mechanism research as a post hoc step, future workflows might intentionally screen not just for selective staining but also immediately apply genetic or biochemical screens to pinpoint why a compound is selective. This could create a feedback loop: knowing the mechanism helps improve the probe or find analogous compounds, which can then be tested again. Such synergy between chemical biology and genomics will likely yield a plethora of new probes each tied to a novel biological insight (e.g., discovering that a particular orphan transporter's expression pattern can be exploited for cell targeting).

In the bigger picture, cell‐selective fluorescent probes are not only imaging tools but also herald potential theranostic applications.^[64]^ A probe that selectively accumulates in a certain pathological cell type (like a cancer stem cell, or a reactive immune cell in an autoimmune disease) could be used to deliver drugs or photosensitizers specifically to those cells, minimizing off‐target effects. Indeed, some protein‐oriented probes (like TiY targeting vimentin in TICs) have shown ability to carry toxic payloads or to be used in fluorescence‐guided surgery.^[65]^ By elucidating mechanisms, we ensure that such translational uses are built on a solid understanding (we know which cells will be hit and why). In conclusion, the landscape of cell‐selective fluorescent probes is rapidly evolving, with each mechanistic class contributing its own perspective on cellular identity. Mechanism elucidation serves as the compass that guides researchers through this landscape—from the initial discovery of an intriguing selective dye through the maze of potential biological interactions to the final destination of a well‐characterized and reliable imaging tool. As we continue to map out transporter networks, enzyme distributions, and biomolecule differences between cell types, we can anticipate the development of ever more refined “smart” probes. These smart probes will not only illuminate specific cells but also shed light on the underlying biology that makes those cells unique. By marrying chemical diversity with biological specificity, the next generation of cell‐selective probes will enhance our ability to visualize, understand, and eventually manipulate complex biological systems with single‐cell precision. The future of this field is bright—quite literally—as we design fluorescent molecules that can navigate the inner workings of cells and report back the secrets that distinguish one cell from another.

## CONFLICT OF INTEREST STATEMENT

The authors declare no conflicts of interest.

## Data Availability

Data sharing not applicable to this article as no datasets were generated or analyzed during the current study.
